# Oil palm residue-based cellulose acetate membranes enhanced with zinc oxide and n-methyl pyrrolidinone for batik wastewater treatment

**DOI:** 10.1186/s40643-025-00880-x

**Published:** 2025-06-19

**Authors:** Belladini Lovely, Hasna Amalia Fauziyyah, Shendy Krisdayanti, Muhamad Zakky Irsyada, Lisna Efiyanti, Wara Dyah Pita Rengga, Novitri Hastuti, R. A. Ilyas, Mohd Nor Faiz Norrrahim, Victor Feizal Knight

**Affiliations:** 1 Research Center for Biomass and Bioproducts, National Research and Innovation Agency (BRIN), Bogor, 16911 West Java Indonesia; 2 Department of Chemical Engineering, Universitas Negeri Semarang, Semarang, 50229 Central Java Indonesia; 3 Department of Chemical Engineering, Faculty of Chemical and Energy Engineering, Universiti Teknologi Malaysia, 81310 Skudai, Johor Malaysia; 4 Centre for Advanced Composite Materials (CACM), Universiti Teknologi Malaysia, 81310 Skudai, Johor Malaysia; 5 Research Collaboration Center for Nanocellulose (BRIN - UNAND), Padang, 25163 West Sumatra Indonesia; 6 Research Centre for Chemical Defence, Defence Research Institute (DRI), Universiti Pertahanan Nasional Malaysia, Kuala Lumpur, Malaysia

**Keywords:** Oil palm empty fruit bunch (OPEFB), Membrane, Cellulose acetate, Zinc oxide (ZnO), N-methyl pyrrolidinone (NMP), Remediation

## Abstract

As the world’s top producers of oil palm (*Elaeis guineensis*), Indonesia and Malaysia are urged to propose a value-added valorization of its lignocellulosic biomass, oil palm empty fruit bunches (OPEFB). Meanwhile, the nations’ signature ‘batik’ textile industries are in dire need of optimum remediation treatments of their wastewater high in harmful dyes and chemicals. Organic–inorganic hybrid systems of mixed matrix membranes (MMMs) for heavy metals removal were prepared using OPEFB-based cellulose acetate (CA) and zinc oxide (ZnO; 0.5, 0.75, 1%, w/v) in N-methyl pyrrolidinone (NMP; 89, 90, 91%, v/v). The high crystallinity (62.42%) and fibrils’ web-like structure of OPEFB-CA were confirmed. Microscopic observation of OPEFB CA-NMP-ZnO membranes evidenced the porous yet smooth surface due to the use of plasticizing NMP, as well as uniform dispersion of ZnO particles. MMM 2 (0.75%ZnO; 90%NMP) was the best-performing membrane mechanically with excellent tensile strength (1.78 MPa), Young’s modulus (0.13 GPa), and elongation-at-break (2.59%), while thermal stability (T_d,5%_, 291 °C) improvement was also highlighted. Pores characteristics on size, volume, and surface area were discussed, too. Remediation performance was excellent even at high (20%) effluent concentration reaching 28% and 65% removal of Cu and Pb, respectively, by MMM 1 (0.5%ZnO; 89%NMP). These findings confirmed the promising prospect of the developed membranes as a wastewater remediation treatment, including in textile industries.

**Graphical Abstract:**

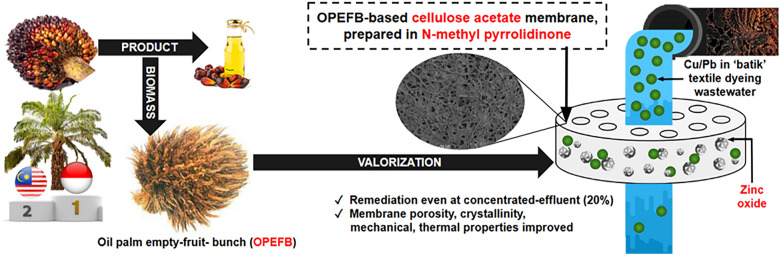

## Introduction

‘Batik’ textile dyeing technique originates from the Indonesian-Javanese and can be traced back to the seventeenth century and is a UNESCO-recognized heritage of Southeast Asia’s Malay nations. ‘Batik’ applies melted wax and resin dye-based intricate designs on a cloth prior to multiple washing and boiling. The generated effluent is typically in large amounts and highly polluted yet handled through minimum to no proper wastewater treatment, risking the environment (Zakaria et al. 2023; Ramakreshnan et al. 2020; Buthiyappan et al. 2016; Rezagama et al. 2020). Even generally, textiles are among the world’s highest effluent-generating sectors (Javed et al. 2025; Slama et al. 2021; Jorge et al. 2023). Though authentic ‘batik’ productions use plant or insect-based dyes, the large-scale demands forced the substitution to the use of various types of synthetic dyes (Slama et al. 2021; Ghaly et al. 2013; Mani et al. 2019; Rashidi et al. 2012). In consequence, the environmental concern possessed by the resulting wastewater is often of significance due to the posed pollutants, not only the excess wax, chemicals, grease, surfactant, silicate, soluble salts, odor, and color, but also suspended/dissolved solids, biochemical oxygen demand (BOD), chemical oxygen demand (COD), and trace heavy metals (e.g., Cu, Pb, Cr, Co, Ni, As, Cd, Zn, Hg). Dyes’ complex molecular structures enable the pollutants to remain in the water for a prolonged time, disrupting the sunlight penetration depth necessary for photosynthesis and re-oxygenation of the existing aquatic, photoautotrophic organisms. Subsequently, the water’s dissolved oxygen (DO) concentration is reduced while BOD rises (Budiyanto et al. 2018; Azha and Ismail 2021; Juliani 2021; Rahmaniah et al. 2019; Lestari et al. 2019; Masupha 2008; Odubanjo et al. 2021; Desa et al. 2019).

To solve such a recurring issue, membrane technology is among the most applied heavy metal removal approaches, including wastewater treatment. Membranes porosity, selectivity, and permeability filter and separate substances by size using pressure when wastewater is pumped through modules. This approach is low-energy and easily operable with minimum to no pollution (Z.-P. Wang et al. 2011; Istirokhatun et al. 2021). According to the retained substances’ particle size, membrane separation can be classified into reverse osmosis, nanofiltration, ultrafiltration, and microfiltration, from the biggest to smallest pore diameter, ranging from 100 nm–10 µm to 0.1–1 nm (Z. Yang et al. 2019). However, these technologies face some prevalent constraints, including the accumulated dissolved solids and dyes when treating complex and highly concentrated effluents, including those of textile dyeing industries. Fouling requires indispensable, sometimes multiple, pre/post-treatments (C.-Z. Liang et al. 2014; Alventosa-deLara et al. 2014; Amin and Nizam 2016). Thus, membrane modifications have been extensively studied to overcome this main limitation, one of which is the introduction of organic–inorganic hybrid membranes (or mixed matrix membranes; MMMs). MMMs combine organic polymer (as the matrix or continuous phase) with inorganic porous ceramics (as the filler or dispersed phase). As one of MMMs’ commonly used inorganic materials, metal oxides are carbon-based and advantageous due to their chemical, mechanical, and thermal stabilities owing to the uniform macro-porous support and meso-/micro-porous barrier layer structure. Their charge transport and specific electronic structure arrangements give them a high adsorption capacity (Jebelli et al. 2024). Metal oxide-driven photocatalysis generates oxidizing species during photon irradiation on the surface of a semiconducting material, which decomposes organic pollutants in wastewater within a short time, is scalable and cost-effective, without persistent by-products (Abdelsamad et al. 2018; Karthik et al. 2024; Shukla et al. 2025; Etafo et al. 2024). Combining these features with polymers is proven to be among the alternatives to intensify the permeability and selectivity, surpassing aging trade-offs empirically observed for the neat-polymeric membranes (Z. Yang et al. 2019; Kamble et al. 2021; Nuhnen and Janiak 2021; Vasanth and Prasad 2019; Tanvidkar et al. 2023).

In selecting polymeric ingredients for MMMs, cellulose is the Earth’s most abundant polymer, naturally biodegradable and extractable from wood, plants, algae, bacteria, and tunicate. Cellulose is easy to modify and compatible, hence its multifaceted applicability, including incorporation with organic/inorganic materials in a hybrid system (Jebelli et al. 2024; Abdelsamad et al. 2018; Karthik et al. 2024). Cellulose acetate (CA), a cellulose ester derivative, is of particular interest to membrane researchers, as evidenced by the spike of interest in the past decade (Vatanpour et al. 2022; Islam et al. 2023). CA excels in its ease of availability, low price, biodegradability, low/non-toxicity, hydrophilicity, chemical–mechanical–thermal stability, water affinity, film-formability, desalting ability, transport properties, design versatility, facile manufacturing, and environmental benignity (Miao et al. 2020; Wei et al. 2020; Fischer et al. 2008; Zugenmaier 2004; Sharma et al. 2012; Syamani 2020; Tahazadeh et al. 2021; Rajeswari et al. 2017; Ghaee et al. 2016; Rana et al. 2022; Febriasari et al. 2024; Jamaluddin et al. 2022). However, pristine CAs suffer from a few vital disadvantages, such as inert functional groups, poor chemical–thermal–mechanical stability, and incompatibility with affinity-based adsorption separation. Compared to hydrophobic membranes, neat-CA membranes provide a slower solvent/coagulant diffusion capacity during phase inversion preparation. This leads to the membrane’s dense skin layer, hence the low porosity, flux, and protein rejection ratio. Pure-CA membranes also operate at a narrow range of pH and temperature (Sivakumar et al. 1999; Zavastin et al. 2010; Arthanareeswaran and Thanikaivelan 2010; Karthik et al. 2024) and are less resistant to microbial corrosion, oxidation, and other organic pollutants (Shenvi et al. 2014; Zhou et al. 2016). Addressing this gap, combining metal oxides and CA in a MMMs system provides antifouling, anti-bacterial/fungal, photocatalytic, and UV-blocking features. Cellulose-based material surfaces act as ideal carriers after their hydrophilic substrates to accelerate nucleation and growth of metal oxide particles onto them. Compared with other popularly reported inorganic additives for membranes (e.g., titanium dioxide (TiO_2_), alumina (Al_2_O_3_)), zinc oxide (ZnO) is superior for cost-effectiveness, low toxicity, and specific surfaces owing to its crystallographic structure (S. Liang et al. 2012; Tam et al. 2008; Alhalili et al. 2021; Hong and He 2014; Asiri et al. 2021; Rashid et al. 2022). ZnO excelled in heavy metal ion removal rate when compared with other alternatives (i.e., a commercial TiO_2_ P25 and a modified version) (Hua et al. 2012; Lee et al. 2005). A low-temperature, low-cost dye removal study also proved ZnO’s unique nano-rod/tube shapes’ influence in superior photocatalytic properties against TiO_2_ and ZnO-TiO_2_ combination (Guo et al. 2011; Sheikh et al. 2020). The reported improvements on ZnO-incorporated membranes include permeation, flexibility, hardness, dye degradation capacity, hydrophilicity, surface roughness, profile valley depth, mechanical, thermal, and chemical stability (Tang et al. 2006; Rajeswari et al. 2017; Alhalili et al. 2021; Asiri et al. 2021; Mahlangu et al. 2017). The essential strong hydrophilicity can be retained by adhering to the hydrophilic functional groups (e.g., –OH, –SO_3_H, –COOH) on top of the chemical bonding between oxide and the pairing polymer (Wu and Xue 2011; Shen et al. 2012, 2020). The promoted hydrophilicity may contribute to fouling mitigation as most foulants are hydrophobic (Vatanpour et al. 2022). The concern regarding ZnO’s toxicity has been debunked as well (Balta et al. 2012; Franklin et al. 2007; Ngulube et al. 2024), confirming its safety. Especially by incorporating ZnO in a carrier (CA), potential leaching could be kept below the standard levels for effluent reuse and discharge, proving its long-term stability (Zhang et al. 2019; Ben Dassi et al. 2021).

The solvent used to prepare CA-based membranes also matters. Compared to other popular contenders (e.g., acetone, dimethylformamide (DMF), or dimethylacetamide (DMAc), tetrahydrofuran (THF)), N-methyl pyrrolidinone (NMP) possesses a myriad of prospects. These include dispersibility, permeate concentration, water flux, sorption selectivity, tensile strength, elastic modulus, transparency, roughness, tailorable porosity, as well as post-use nodule/void sizes/shapes and surface morphology. NMP’s volatility or vapor pressure was evidenced to improve CA chains relaxation and membrane formability, with the plasticization effect via the increased free volume of CA chains. NMP’s desired boiling point also highly influences thermal stability (Tabe-Mohammadi et al. 2001; van den Berg et al. 2007; Cindradewi et al. 2021; Lee et al. 2018). The potentials of CA-acetone-ZnO (Rajeswari et al. 2017; El-Noss et al. 2020; Durthi and Rajulapati 2018; Hassan et al. 2017), CA-DMF-ZnO (Asiri et al. 2021; Alhalili et al. 2021; M. Ali et al. 2011; Chaurasia et al. 2010; H. S. Hassan et al. 2017), CA-DMAc-ZnO (Hassan et al. 2017; Zhou et al. 2023; Nasouri 2019; Suwantong and Supaphol 2015), and CA-THF-ZnO (Douna et al. 2023) systems have been studied; however, CA-NMP-ZnO has yet to be explored to date especially for wastewater remediation, implying its novelty.

Speaking of the cellulosic source in preparing CA, agricultural wastes have become among the most extensively studied renewable options. Cultivated mainly for crude palm oil, oil palm (*Elaeis guineensis*) is a tropical-perennial oleaginous crop that grows along equatorial Southeast Asia, South America, and Africa (Wahid et al. 2005; Corley and Tinker 2008). Despite originating from West/Central Africa (Mutsaers 2019), Indonesia, Malaysia, and Thailand are named as today’s top producers, where 2024 global production surpassed 80 million MT (United States Department of Agriculture 2024). Oil palm has exceptional productivity (36% of global oils while taking merely 8% of the oil croplands), yield (13, 8, and 7 times greater than soybean, sunflower, and rapeseed, respectively), and economic life span (25 years) (Ritchie et al. 2023; Chang 2014; Tye et al. 2011). In line with such unprecedented growth, a vast quantity of waste is generated every year. Per T CPO, half of the generated solid wastes (~ 1.1–1.5 T) is a residue acquired post-sterilization and stripping of the fresh fruit bunches, called oil palm empty-fruit-bunch (OPEFB) (Dahnum et al. 2015; Dalimin 1995; Loh 2017). OPEFB is characterized by its brown color, hard/tough structure, bulk density, low calorific value, and high contents of moisture (> 60% of total weight), ash, and mineral matter, ultimately leading to its low conversion efficiencies and economic value (Sulaiman et al. 2010; Ahmad et al. 2019; Hassan et al. 2010; Chang 2014). OPEFB utilization has been limited to low-value-added applications such as mulch, fertilizer/compost, manure, and cultivation substrate, whereas direct burning may cause incomplete combustion and the release of fine ash or white smoke, or even worse, fouling and pest attraction when left unattended; displaying a serious disposal challenge (Geng 2013; Kerdsuwan and Laohalidanond 2011; Yusoff 2006; Law et al. 2007). Fortunately, OPEFB holds vast potential as it contains an exceptionally high amount of cellulose (40–65%) while not disrupting the security of lignocellulosic sources intended for food (corn, soybean, coconut) or forest/plantation (wood, cotton) (Hornung 2014; Yusoff 2006; Abdullah and Sulaiman 2013; Razali et al. 2017). OPEFB-derived cellulose is also proven to be remarkably high in purity (Megashah et al. 2018). However, despite the great prospect, OPEFB utilization in cellulose acetate-based MMMs has yet to be studied. Hence, OPEFB makes as an attractive and novel cellulosic material, particularly for the development of CA-NMP-ZnO-based membranes. The use of such vastly available, underutilized, and locally sourced biomass for the nations’ signature ‘batik’ wastewater treatment highlights this work’s significance. The effect of NMP and ZnO addition was hypothesized to promote the final functional properties of CA membrane, not only the heavy metal remediation performance, but also the physical, mechanical, morphological, and thermal stability.

## Materials and methods

### Materials

Oil palm empty-fruit-bunch (OPEFB) was provided by a local, traditional farm in Cibadak, West Java, Indonesia. Distilled water, ethanol, glacial acetic acid (CH_3_COOH), sulfuric acid (H_2_SO_4_, 98%, v/v), acetic anhydride (C_4_H_6_O_3_), N-methyl-2-pyrrolidone or NMP (C_5_H_9_NO), zinc oxide (ZnO), dimethyl sulfoxide or DMSO (C_2_H_6_OS), sodium hydroxide (NaOH), hydrochloric acid (HCl), and phenolphthalein (C_20_H_14_O_4_) were purchased from Merck & Co., Inc. (Rahway, NJ, USA). Commercial-grade cellulose was from Sigma-Aldrich (St. Louis, MO, USA).

### Cellulose acetate (CA) preparation

Cellulose acetate (CA) was prepared from oil palm empty-fruit-bunch (OPEFB) dry pulp whose moisture content was tested using a MOC63u moisture analyzer (Shimadzu Corporation, Kyoto, Japan) before the extraction. The activation process was begun by dissolving OPEFB pulp (5 g) into glacial acetic acid (150 mL) before being continuously stirred (1500 rpm, 38 °C, 3 h). Next was the acetylation step by adding acetic anhydride (45 mL) and 98%-sulfuric acid (1 mL) as catalyst and back being stirred (1500 rpm, 25 °C, 2.5 h). For the hydrolysis stage, distilled water (6 mL) and glacial acetic acid (15 mL) were added to stop the acetylation, then stirred again under low heat (1500 rpm, 50 °C, 30 min). To start the sedimentation process, the mixture was centrifuged (4000 G, 25 °C, 10 min) in a 50 mL tube to separate the pellet from the supernatant. The resulting pellet was extracted using filter paper before drying in the oven (50 °C, 6 h). Identical steps were conducted using commercial cellulose to prepare commercial CA.

### Membrane preparation

Cellulose acetate (CA) membrane was prepared using the synthesized CA (5%) and three different variations of added NMP and ZnO, respectively (Table 1). Firstly, CA (0.5 g) was dissolved in dimethyl sulfoxide (DMSO; 10 mL). NMP was diluted in distilled water based on the desired concentration, while ZnO was prepared in DMSO (10 mL). Afterward, the diluted NMP and ZnO were added into CA and stirred under low heat (60 °C, 3 h). The solution was left to sit for an hour at room temperature to remove air bubbles. The membrane was prepared using a solution casting technique where the mixture was poured into a petri dish and dried (55 °C, 20 h) to let NMP (solvent) evaporate.

**Table 1:** Content of different CA membrane types

OPEFB CA-NMP-ZnO membranes	NMP (%, v/v)	ZnO (%, w/v)
MMM 1	89	0.50
MMM 2	90	0.75
MMM 3	91	1

### Crystallinity

The relative crystallinity of cellulose and CA was investigated using SmartLab (Rigaku Corporation, Wilmington, MA, USA) X-ray diffraction (XRD), which produced spectra that plot X-ray diffraction angle (2θ) vs intensity. The popularly easy and time-efficient Segal's “height XRD” method (Segal et al. 1959) was used to determine the crystallinity index (CrI), which measures the intensity ratio between the crystalline and amorphous peaks in the resulting spectra. The formula used is below, where I_am_ is the minimum intensity of the amorphous peak (or the maximum point where the entirely amorphous region is located) at around 2θ = 18°–19°, and I_total_ is the maximum intensity of the crystalline/major peak at around 2θ = 22°–23° (Nurhadi et al. 2022; Park et al. 2010).

$$CrI (\%)=1-\frac{{I}_{am}}{{I}_{total}}$$

### Morphological and elemental analysis

The surface morphology and elemental distribution of cellulose, CA, and ZnO were characterized by the NeoScope JSM-6510LV (JEOL Ltd., Tokyo, Japan) Scanning Electron Microscope–Energy Dispersive X-ray Spectroscopy (SEM–EDS), producing SEM micrographs and EDS mapping, field-of-view images, and energy vs. intensity spectra. It was then mounted onto a pin stub mount using copper tape before being coated with gold at 20 mA. Specimens were subsequently imaged under a high vacuum at different operating voltages and magnifications: 3 kV and 50–1000× for SEM; and 15 kV and 1000× for EDS.

### Porosity

The membranes’ adsorption–desorption isotherms and pore characteristics (surface area, volume, and radius) were determined with a NOVA-4200e surface area and pore size analyzer (Anton Paar QuantaTec Inc., Boynton Beach, FL, USA) at degassing temperature of 120 °C (3 h) using the Brunauer–Emmett–Teller (BET) and Barrett–Joyner–Halenda (BJH) methods.

### Mechanical properties

The mechanical testing was measured according to ASTM D638-22 (ASTM International 2022) with Universal Testing Machines (UTM) AGS-X (Shimadzu Corporation, Kyoto, Japan). The membrane’s tensile strength was measured with 5 kN preload at a test speed of 5 mm/min until the films (2 × 5 cm) were ruptured and calculated from the ratio of maximum load to the cross-sectional area.

### Thermal property

Thermogravimetric analysis (TGA) was performed using a Simultaneous Thermal Analyzer (STA) 6000 (PerkinElmer, Shelton, CT, USA) that plots temperature vs weight change. The sample (5–15 mg) was spread in the pan to maximize surface area. It was then ramped at 10 °C/minute between 20 and 500 °C.

### Remediation performance

The concentration of heavy metals of copper (Cu) and lead (Pb) in the liquid waste was determined using a PinAAcle 500 Flame Atomic Absorption Spectrometer (AAS) (PerkinElmer, Shelton, CT, USA) at a pressure of 1 bar. The effluent of a local ‘batik’ factory wastewater in different concentrations (10, 15, and 20%) was employed.

## Results and discussion

### Crystallinity

**Fig. 1:**
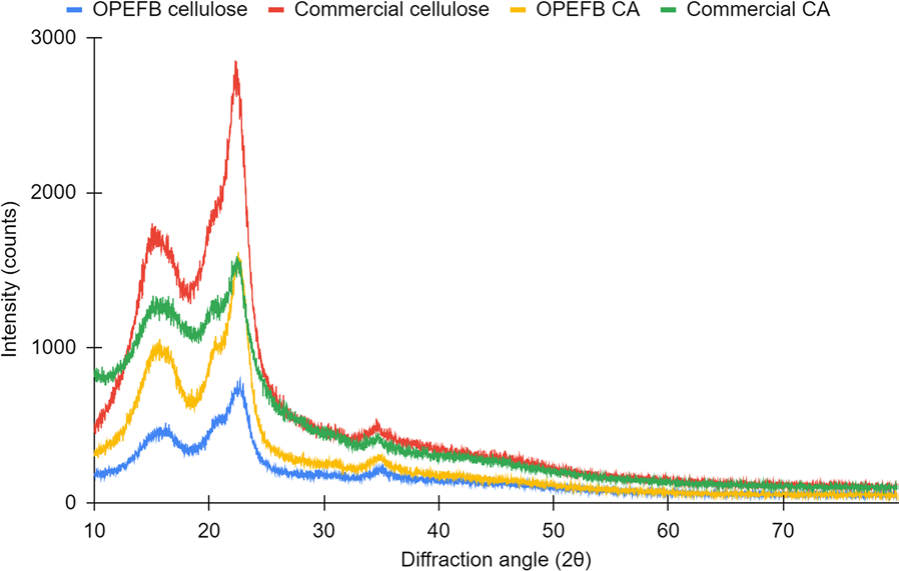
XRD diffraction spectra of cellulose and CA

Figure 1 presents the diffraction peaks of cellulose and CA. The crystallinity index (CrI) is measured by determining two distinct peaks: 1) the sharp, high, and obvious major or crystalline or monoclinic peak, namely I_total_ at 2θ = 20°–25°, and 2) the broad secondary or amorphous peak, namely I_am_, around 2θ = 12°–20°. While some minuscule peaks are likely assigned to the crystalline phase of residual salts, impurities, or trace elements, all of the arising major peaks in both OPEFB samples (cellulose and CA) could be ascribed to the characteristic patterns of the Cellulose I type. The aforesaid peaks are the Miller indices of (1Ī0) at 2θ = 15°, (110) at 2θ = 18°, (200) at 2θ = 22.5°, and (004) at 2θ = 35° (Liu et al. 2022). This confirmed the successfully retained crystallinity profile and structure of the native cellulose contained in the rawest form as found in nature, despite the acetylation process subjected when synthesizing OPEFB CA.

CrI of all cellulose and CAs is presented in Table 2. CrI is defined as the mass ratio of the crystalline substance in the total dry sample based on the resulting XRD crystallographic two-phase model (Daicho et al. 2018). The preservation of high crystallinity was also confirmed in its CrI value of 62%. Unlike commercial CA, the effect of acetylation is apparent in its much lower CrI value of 34%. This is due to the substituting acetyl group, which eventually widened the interfibrillar distance and spaces between crystal facets and caused microfibrillar structure breakdown. (Daud and Djuned 2015; Fan et al. 2009; Kono et al. 1999; Barud et al. 2008; Filho et al. 2000). The promoted amorphous nature can be seen in the high intensity of the amorphous peak (I_am_ at 2θ = 19°) of the commercial CA spectra. This is related to the ‘post-acetylation disorder’s peak of CAs at 2θ = 8°, often cited as the principal characteristic of semicrystalline acetylated derivative cellulose. These phenomena are identical to other CA reports, including the OPEFB-derived ones (Daud and Djuned 2015; Djuned et al. 2014). This finding confirmed the remarkable prospect of OPEFB being utilized as a raw material in synthesizing cellulose acetate with a maintained high crystalline.

**Table 2:** Crystallinity index (CrI) of cellulose and CA

Sample	Amorphous peak		Crystalline peak		CrI (%)
	2θ	I_am_	2θ	I_total_	
OPEFB cellulose	18.11	312	22.72	794	61
Commercial cellulose	18.24	1314	22.40	2848	54
OPEFB CA	18.05	608	22.57	1618	62
Commercial CA	19.11	1028	22.76	1556	34

### Morphological and elemental analysis

**Fig. 2:**
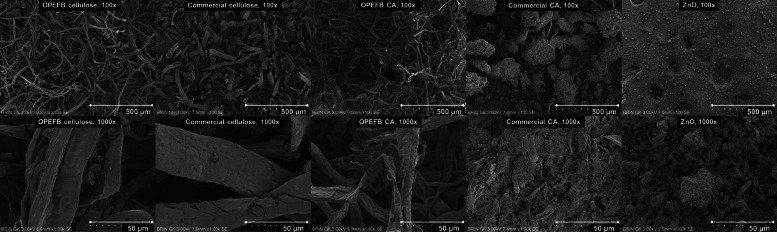
SEM images of cellulose, CA, ZnO: ×100 (500 µm scale), ×1000 (50 µm scale)

Figure 2 displays the surface SEM images of cellulose and CA. The sole difference between the two cellulose samples was the shorter/thicker microfibrils of commercial cellulose, possibly due to the source or extraction method. For example, cellulose extracted from sea plants and animals provides a significantly higher aspect ratio than those derived from wood and cotton (Panchal et al. 2018). OPEFB-derived cellulose itself displayed distinctive rod-like long microfibrils (Lai et al. 2021). Harsher pretreatments during cellulose extraction might lead to more intense dissolution of amorphous domains and longitudinal cutting of the microfibrils (Kalia et al. 2011). Both cellulose samples showed the appearance of numerous microfibrils, some of which are still strongly entangled and form thick bundles but with overall smooth surfaces. The smooth surface area of microfibrils was the outcome of the bleaching and delignification process that washed off hemicellulose, lignin, and other impurities from the raw OPEFB fiber (Susi et al. 2023).

In contrast, the surface of OPEFB-CA microfibrils appeared rougher, indicating the acetylation influence. The disrupted fibril–fibril hydrogen bonding as the aftermath of acetyl group introduction substituting hydroxyl group was also evidenced in the observed detachment of CA microfibrils from each other. However, a web-like structure was created, nonetheless. Unlike the CA prepared using the aforesaid commercial cellulose, due to the observed shorter/thicker microfibrils on top of the subjected acetylation process, commercial CA micrographs displayed phenomena of substantial agglomeration/aggregation of the microfibrils. The formed fibril bundles were non-uniform in size and shape, leaving massive voids between one another. Synthesis of cellulose derivatives like CAs provided sophisticated changes to the network structures through aggregation-induced phase separation, impacting the final microstructure and rigidity. The mass-heat transfer during film casting might also lead to the thermodynamic instability of cellulose-based materials, and phase separation occurs (Ferrarezi et al. 2013; Lee et al. 2006). These findings provided an insightful look into OPEFB's success in providing a desirable morphological trait, namely the highly fibrillated and web-like network in cellulose and CA synthesis, especially for heavy metal remediation membrane applications. Figure 2 also presents the SEM image of pristine ZnO particles prior to their incorporation into the CA system during membrane preparation. ZnO’s spherical shape in nature was confirmed, as well as the agglomerates/aggregates and uneven separation (Muhammad et al. 2019; Mohan and Renjanadevi 2016). Its effectiveness as a filler in a hybrid organic–inorganic CA-based membrane system is further discussed.

**Fig. 3:**
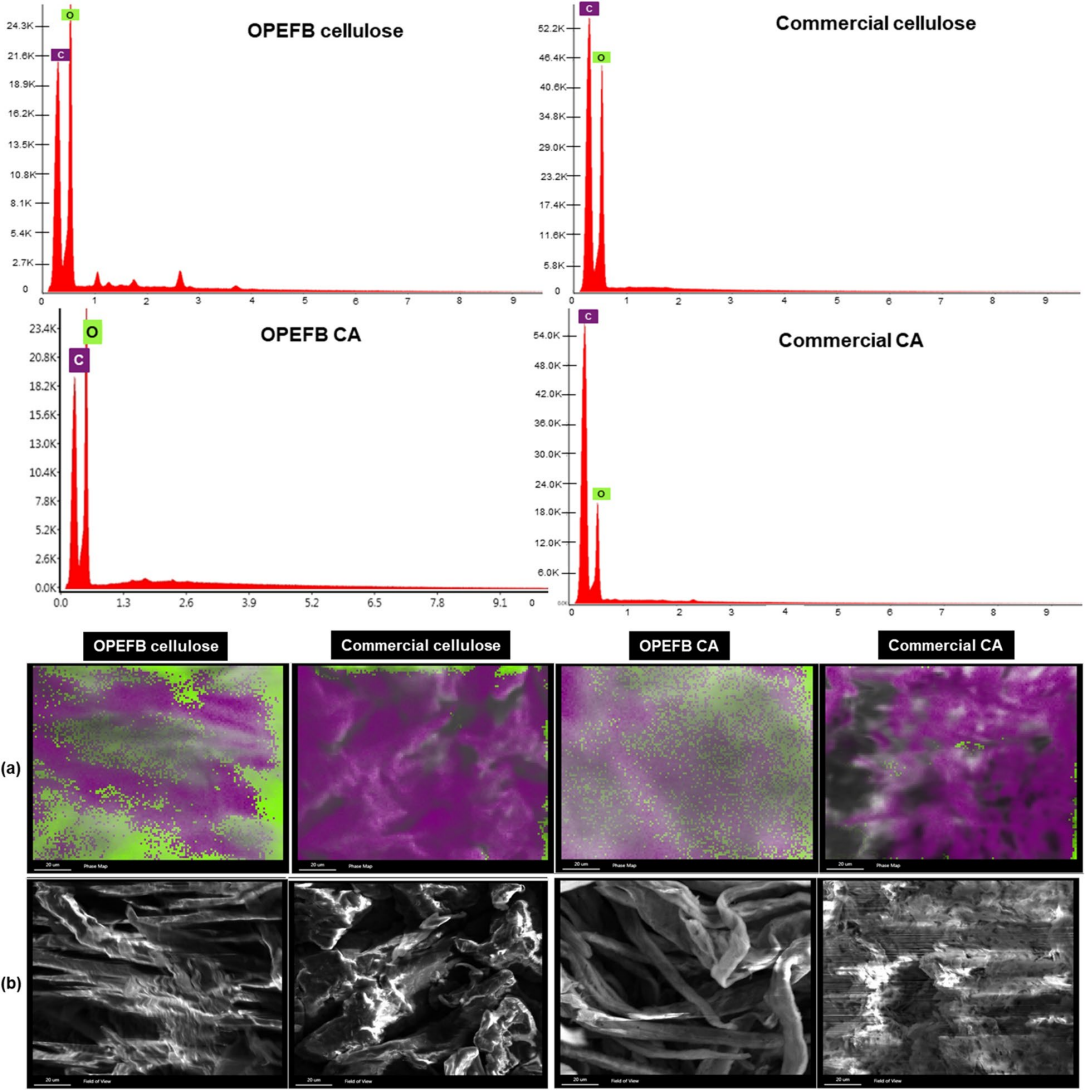
EDS spectra and images (×1000, 20 µm scale): **a** field-of-view and **b** mapping of cellulose and CA

To support the morphological findings, Fig. 3 showcases the EDS spectra for elemental analysis. Carbon (C) and oxygen (O) peaks were apparent in the spectra of both OPEFB-based and commercial cellulose. Once processed to be CAs, both cellulose spectra showed an appearance of the same elements, C and O, but with lower intensities. This shift proved the acetylation effect which reduced the number of both elements after the acetyl group introduction to replace the hydroxyl group of neat cellulose. The only difference between the spectra of OPEFB-based and commercial samples was the O peak being more intense than the C peak for OPEFB-based specimens. Meanwhile, the opposite was seen for commercial ones, as similarly found in another pristine CA study (Durthi and Rajulapati 2018). Different cellulose sources may offer different structural properties (Seddiqi et al. 2021). While for CA, the probable cause was the number of hydroxyl groups of cellulose repeat units replaced by acetyl groups. These elemental observations were further confirmed visually by the EDS field-of-view and mapping images (Fig. 3a, b) as a relation between chemical composition and structure, especially for membrane materials (Corneal et al. 2010; Qin et al. 2015).

**Fig. 4:**
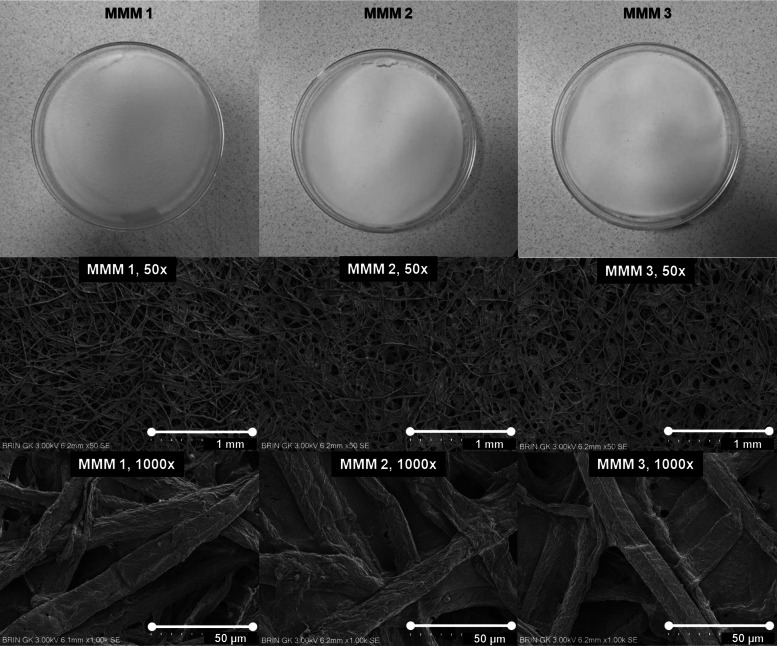
Naked-eye detection and SEM images of OPEFB CA-NMP-ZnO membranes: ×50 (1 mm scale), ×1000 (50 µm scale)

Furthermore, the prospect of the ZnO-administered OPEFB CA system was discussed by analyzing Fig. 4**.** Through naked-eye detection, overall, all three CA-NMP-ZnO membranes looked identical, therefore, micrographic morphological observation was conducted next. As the solvent of choice, NMP possessed low volatility or vapor pressure. Its slow evaporation allowed the CA chains to undergo a lengthy relaxation process to form a membrane with a stable structure and well-packed configuration. The plasticization effect was also provided by NMP as it retained longer in the system and aided in weakening the CA region, forming porous regions (Lee et al. 2018; Tabe-Mohammadi et al. 2001). It can be noted through investigating the random pore distribution that the prepared membrane could be classified as an asymmetric membrane (Rajeswari et al. 2017). Despite the visible pinholes, cracks, and close-cave type voids, NMP-based CA membranes were proven to perform desirable separation ability with high selectivity through the walls of the close-cave structural model (Tabe-Mohammadi et al. 2001). The membrane surface smoothness was an improvement compared to those prepared with other more volatile solvents (e.g., acetone), including the after-use observation where the membrane shrinking and the visible voids disappearance were observed (Tabe-Mohammadi et al. 2001; W. G. Lee et al. 2018; Cindradewi et al. 2021), confirming the NMP-based membranes’ longevity. Whereas, regarding ZnO, such inorganic material incorporation could affect thermodynamic/kinetics and increase suspension viscosity, yet at an excess amount might also decrease non-solvent diffusion and suppress the pores formation (Asiri et al. 2021; Shen et al. 2020; Alhalili et al. 2021; Balta et al. 2012), thus the optimum ratio is vital. ZnO particles could be seen blended and dispersed along the membranes’ surface due to their hydrophilicity enhancer effect. Particularly at MMM 2 (0.75%ZnO; 90%NMP), the vastly interconnected network between blended ZnO and cellulose fibrils created more cavities/voids and increased roughness compared to MMM 1 (0.5%ZnO; 89%NMP), or essentially more pores (higher porosity) in smaller sizes and overall surface area (El-Noss et al. 2020; Chaurasia et al. 2010; Rajeswari et al. 2017; Gebru and Das 2017). However, at MMM 3 (1%ZnO; 91%NMP), ZnO clusters could be seen due to self-agglomeration/aggregation tendency after the naturally high surface energy of metal oxides (Liu et al. 2021a, b; Barnes et al. 2013), subsequently increasing pore sizes, as seen in other CA-metal oxide membrane studies (Ali et al. 2011; Alhalili et al. 2021; Gebru and Das 2017). A more in-depth investigation of membrane pores analysis is in the next section.

### Porosity

**Fig. 5:**
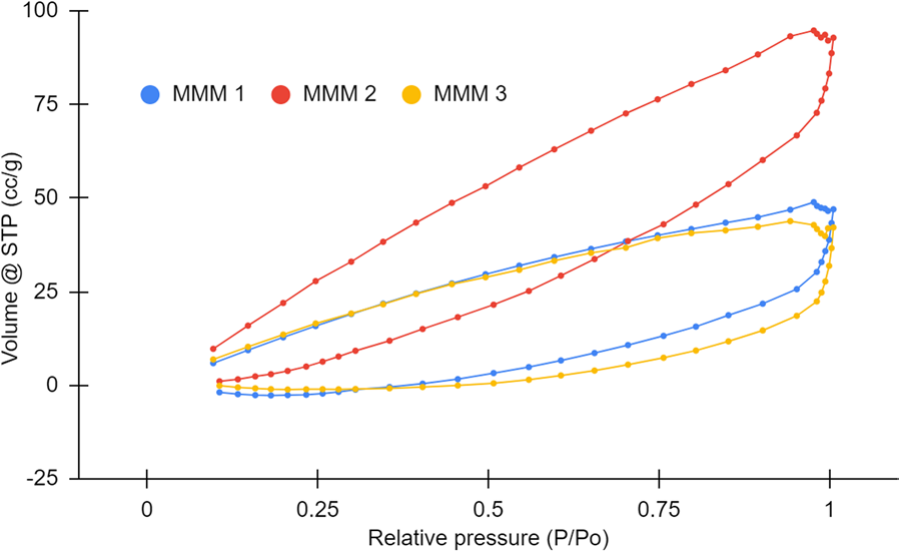
Adsorption–desorption isotherm of OPEFB CA-NMP-ZnO membranes

In terms of membrane pores analysis, BET measures the adsorption aspect of adsorptive material relative to pressure. In this work, such findings would be crucial in determining the remediation performance of the fabricated OPEFB CA-NMP-ZnO membranes on its effectiveness in treating textile dyeing wastewater full of harmful dyes/chemicals. Pores quantitative analysis also would confirm the previously discussed morphological findings. Figure 5 shows the adsorption/desorption isotherm, explaining the relation between the relative pressure (P/P_o_) and specific amount adsorbed (volume at standard temperature and pressure (STP) (cc/g)). The plots showed that the adsorption isotherm type may be measured as mixed types of I and IV (Tien 1994), similar to other CA membrane studies (Nemr et al. 2017; Ragab et al. 2023; Ali et al. 2024). This finding corresponds to IUPAC categories of micro-porosity (≤ 2 nm) and meso-porosity (2–50 nm) (Sing 1985), confirmed by pore statistics in Table 3 that the membranes’ average radius ranged from 1.6 to 2.5 nm, comparable to other reports (Arthanareeswaran and Thanikaivelan 2010). Moreover, the obtained pore sizes were remarkable even compared to that of commercial membranes made of CA (Alves et al. 2020), (both conventional and modified) polyvinylidene difluoride (PVDF), and polyether sulfone (PES) (Yang et al. 2022). As observed morphologically by SEM (Fig. 4), MMM 2 (0.75%ZnO; 90%NMP) indeed possessed the smallest adsorption radius (1.6 nm), while MMM 3 had the largest (1%ZnO; 91%NMP) (2.5 nm). This evidenced the influence of optimum ratio and excessive addition of ZnO, as seen in other cases of metal oxide-doped CA membrane (Arthanareeswaran and Thanikaivelan 2010). Although, across different membranes, pores became smaller during desorption, where all showed identical desorption radius (1.7 nm). Similar to another modified CA membrane report (Ali et al. 2024), the aforementioned superior amount of formed pores and great surface area of MMM 2, as a positive impact of ZnO addition previously discussed in morphology analysis (Fig. 4), was confirmed with the acquired adsorption/desorption isotherm finding.

**Table 3:** Porosity of OPEFB CA-NMP-ZnO membranes

Membrane		Radius, Dv(r) (nm)	Volume (cc/g)	Surface area (m^2^/g)
MMM 1	Adsorption	1.9	0.08	30.7
	Desorption	1.7	0.06	39.1
MMM 2	Adsorption	1.6	0.15	72.1
	Desorption	1.7	0.12	80.8
MMM 3	Adsorption	2.5	0.06	16.7
	Desorption	1.7	0.05	35.2

### Mechanical properties

**Fig. 6:**
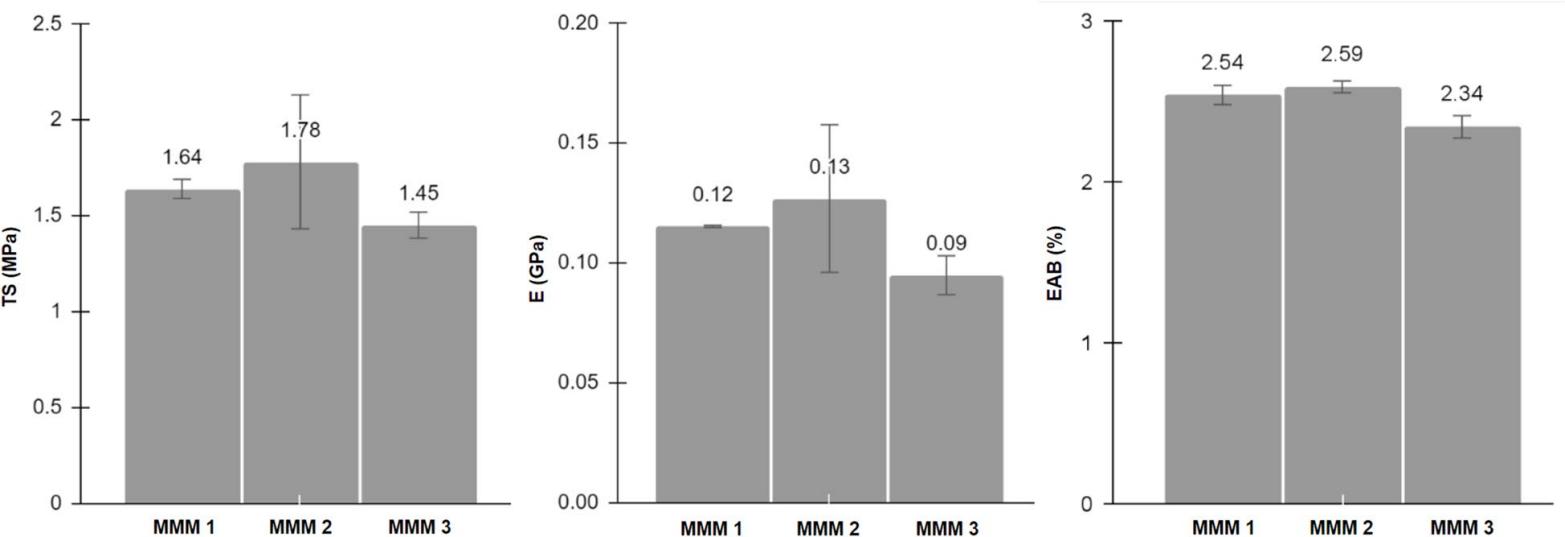
Mechanical properties of OPEFB CA-NMP-ZnO membranes: tensile strength (TS), Young’s modulus or (E), elongation-at-break (EAB)

The effect of varying ratios of OPEFB CA-NMP-ZnO membranes on their mechanical properties is showcased in Fig. 6. MMM 2 (0.75%ZnO; 90%NMP) was the membrane with the best mechanical performance with the highest TS, E, and EAB valued at 1.78 MPa, 0.13 GPa, and 2.59%, respectively. This could be due to the high crystallinity of CA whose chains turn orientation in the direction of the stress when stretched, known as ‘necking’ (Pittarate et al. 2011; Ramakrishna 2005). It could also be due to the improved dense-layer thickness, CA–ZnO interaction, and the macro-voids suppression after the administered ZnO (Arthanareeswaran and Thanikaivelan 2010; Arthanareeswaran et al. 2008; Moghadassi et al. 2014). In contrast, the decline in all three mechanical properties, beginning at MMA 3, proved the effect of agglomerated excess NMP and ZnO and their eventually decreased distribution/dispersion within the CA structure as the matrix, acting as weak links. This phenomenon was similarly investigated in other doped CA membrane studies (Cindradewi et al. 2021; Wibowo et al. 2006; Arthanareeswaran and Thanikaivelan 2010; Arthanareeswaran et al. 2008). Such an influence of ratio and solvent on the mechanical stability of CA membranes has been reported (Stylianopoulos et al. 2012). Regardless, the obtained TS, E, and EAB values are comparable to those of previously researched CA membranes (Arthanareeswaran and Thanikaivelan 2010; Ghaffarian et al. 2015; Arthanareeswaran et al. 2008; Moghadassi et al. 2014).

### Thermal property

**Fig. 7:**
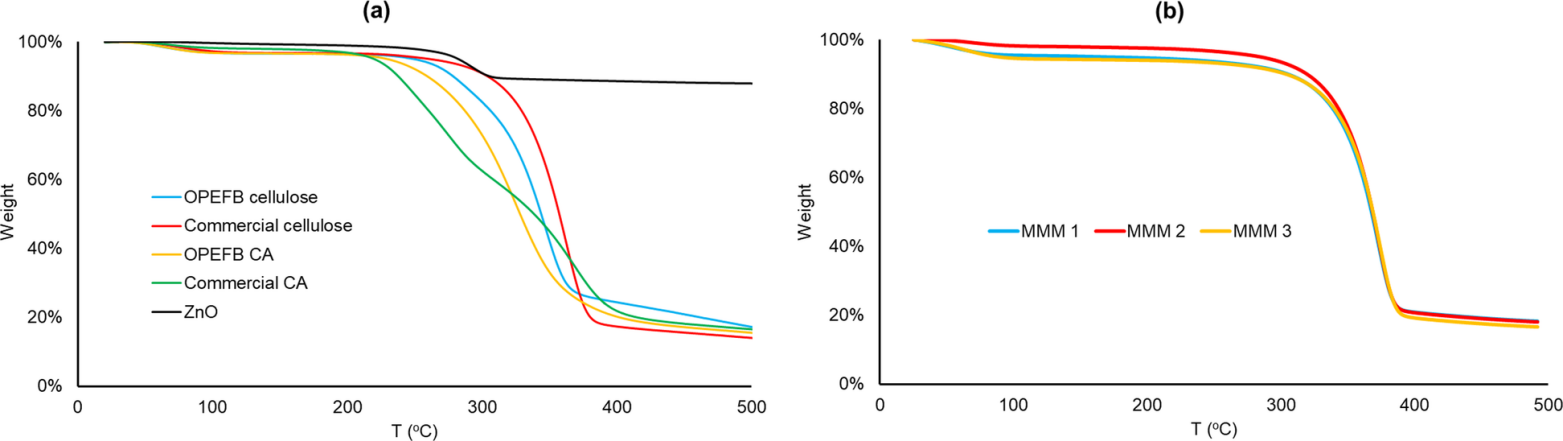
TGA spectra of **a** cellulose, CA, ZnO; **b** OPEFB CA-NMP-ZnO membranes

Thermal stability is crucial when designing wastewater treatment membranes with a long-life span, especially when separating high-temperature (> 60 °C) streams (Zuo et al. 2020). Thermogravimetric findings through weight/mass (loss/gain) as a function of temperature/time could also be insightful in determining decomposition behavior and degradability (Brunšek et al. 2023). Figure 7 plots weight loss over changing temperature. Based on the produced TGA spectra Table 4 lists the samples’ T_d,5%_, or the temperature at a 5%-weight loss, and %-weight at a final testing temperature of 500 °C.

**Table 4:** Degradation temperature and weight loss profile

Sample	T_d,5%_ (^o^C)	%W at 500 °C
OPEFB-cellulose	256	17
Commercial cellulose	270	14
OPEFB CA	235	16
Commercial CA	223	17
Pure ZnO	285	88
MMM 1	213	18
MMM 2	291	18
MMM 3	105	16

The first content decomposed was the absorbed or loosely bound water and any volatile matter starting at temperatures ranging from 40–47 °C to 100 °C through evaporation, dehydration, or volatilization. However, the weight change was typically the least significant. The second yet most significant decomposition stage was on glucose rings in the cellulose chain and acetate functional groups for CAs, through pyrolysis at 260–360 °C leaving char/ash. The slow, final thermal-oxidative decomposition occurred at around 400 °C, marking the degraded elements’ carbonization to ash (Yang et al. 2019; Ilyas et al. 2018; Abral et al. 2019; Araújo et al. 2018; Asrofi et al. 2018; Zhao et al. 2013; Liu et al. 2021a, b; Ali et al. 2011; Cindradewi et al. 2021). As also seen in a previous study (Fauziyyah et al. 2024), both OPEFB and commercial CAs (223–235 °C) were proven to thermally degrade faster than neat cellulose (256–270 °C), as shown by their lower T_d,5%_. Such a decrease in thermal stability was due to the weakened hydrogen bonding post-acetylation as the hydroxyl groups were replaced by acetyl groups. There was also an influence from the crystalline structure fusion, random distribution of substitution (acetyl) groups, and chain low regularity or weak packing, making it thermally weaker (Barud et al. 2008).

In contrast, neat-ZnO powder, being an inorganic material with strong covalent bonds and lacking carbon/hydrogen atoms, performed distinctly superior thermal stability compared to any of the organic, cellulose-based specimens (Malakhov and Samsonov 1966; Johns et al. 1962). It is proven by not only its highest T_d,5%_ compared to all other samples but also most of its weight even at 500 °C (88%), unlike cellulose and CAs, whose %-weight only remained at 14–17% at the same temperature. Thus, ZnO incorporation into the CA system improved the thermal stability of the membranes (Asiri et al. 2021). In addition, as a solvent of choice, NMP possessed high thermal stability due to its high boiling point (Cindradewi et al. 2021). Hence, the remarkably high T_d,5%_ of MMM 2 (0.75%ZnO; 90%NMP) at 291 °C, since a strong CA-ZnO interaction required higher decomposition energy (A. Islam, Yasin, and Rehman 2014; Kim et al. 2010). The obtained value was comparable to that of a highly loaded CA-ZnO (10%) study (Alahmadi and Hussein 2022). This proved the positive thermal impact of not only ZnO incorporation, as seen in other CA-ZnO reports (Pérez-Silva et al. 2023; Alahmadi and Hussein 2022), but also the use of NMP. On the other hand, the extremely low T_d,5%_ of MMM 3 (1%ZnO; 91%NMP) at 105 °C was probably after the excess amount of added ZnO, causing particle agglomeration. This was similarly observed in other studies showing a negative effect on thermal stability by excessive ZnO addition in polypropylene (> 1%) (Esthappan et al. 2015) and polymethylmethacrylate (> 1%) (Hammani et al. 2018).

### Remediation performance

**Fig. 8:**
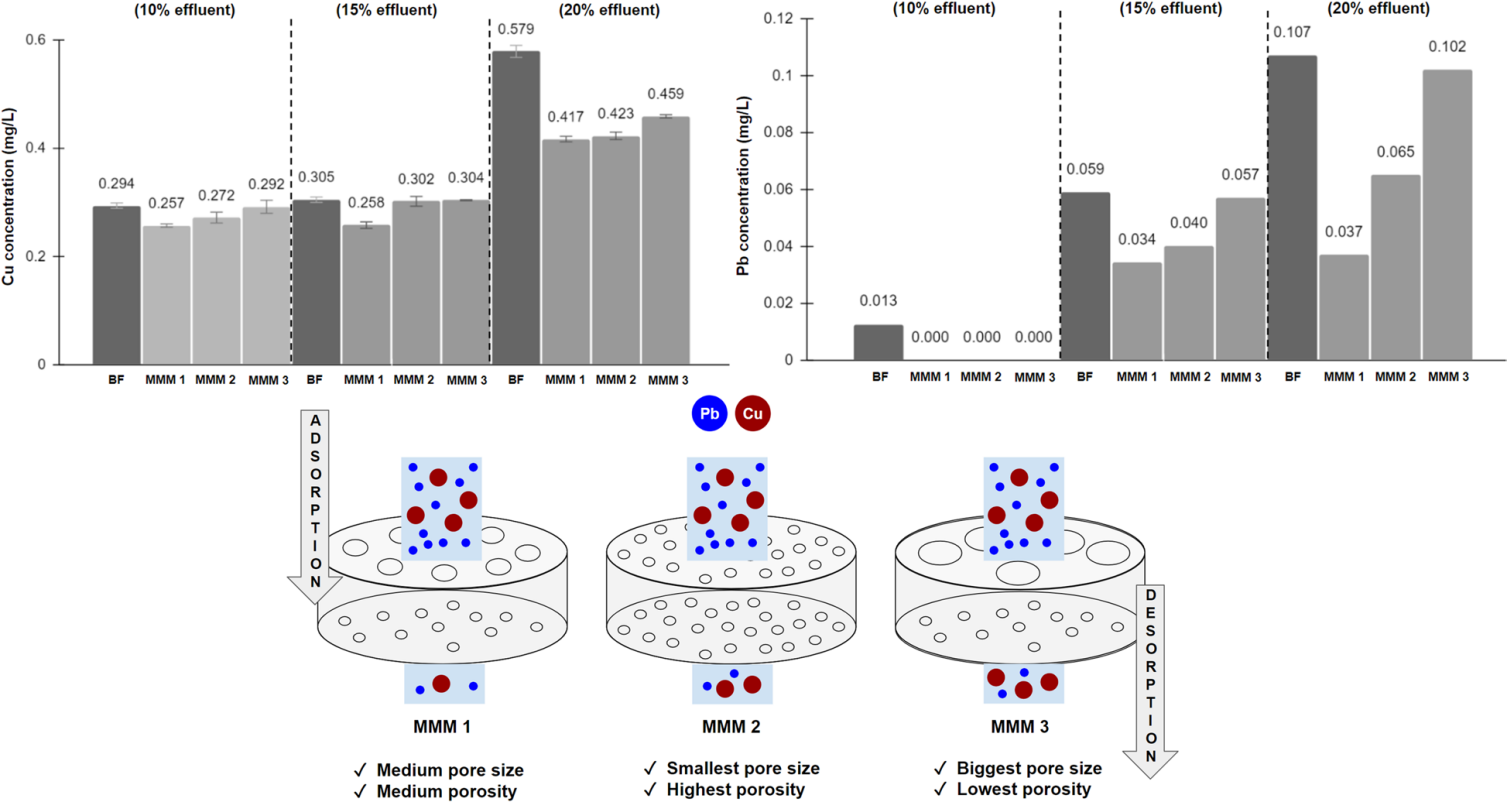
Cu and Pb remediation performance of CA-NMP-ZnO membranes at varying effluent concentrations (BF: Before Filtration), and its illustration

The removal efficiency of Cu and Pb contained in wastewater in varying effluent concentrations by using CA-NMP-ZnO membranes, also referring to each of their porosity (Table 3), are highlighted in Fig. 8. Chemically, hydroxyl groups are the functional groups responsible for heavy metal ions adsorption by CA-based membranes (Liu and Bai 2006). Moreover, peaking of physical adsorption, membrane permeability was raised to a specific limit before lowering when the concentration of filler exceeded, marking the compactness occurring in the membrane structure (Idress et al. 2021; Cong et al. 2010). Generally, at lower effluent concentration (10%), it reached a complete remediation of Pb for all membranes. For both metal ions, the membranes performed exceptionally even at higher effluent concentrations (20%), as investigated in another report (Ji et al. 2012)**,** where the amount of adsorbed heavy metal rose with the increase of the equilibrium concentration.

Interestingly, remediation performance generally dropped with the increase of NMP/ZnO content, possibly due to thickening issues. Such an accumulation led to difficulty for the Cu or Pb to diffuse within the internal and surface pores of the matrix (Gebru and Das 2017). This highlighted the collective influence of not only the membrane’s pore characteristics (radius, volume/porosity, surface area) but also the size of the heavy metals. In general, removal efficiencies of the membranes were slightly superior for Pb compared to Cu, as reported in other cellulose-based membrane studies (Zhou et al. 2004; Ji et al. 2012; Gebru and Das 2017), possibly due to the bigger size/radius of Pb (Pb, 175 pm; Pb^2+^, 119 pm) compared to Cu (Cu, 128 pm; Cu^+^, 77 pm; Cu^2+^, 73 pm) (Wells 1976). The trend was identical to that of a CA-TiO_2_ membrane study (Gebru and Das 2017). The membrane pore characteristics mattered less when it came to Cu removal due to its smaller size, as shown by the almost identical remediation performance across three different membranes. In Pb removal, MMM 1 (0.5%ZnO; 89%NMP) was the best-performing membrane regardless of the effluent concentration, whose removal effectiveness reached 28% and 65% for Cu and Pb, respectively, at 20% effluent. Despite its smallest adsorption pore size (1.6 nm) among all membranes, MMM 2 (0.75%ZnO; 90%NMP) might possess too high of porosity, leading to heavy metal particles passing through the membrane too easily, resulting in the worsened remediation performance. Therefore, MMM 1, with its higher pore size (1.9 nm) yet optimum porosity, allowed effective adsorption of the heavy metals within the membrane network, hence its remarkable removal ability. The agglomerated ZnO at MMM 3 (1%ZnO; 91%NMP) hindered the diffusion of metal ions within the membrane system, hence the worst removal ability, as expected (Gebru and Das 2017).

It is worth noting that following the superior pore sizes (Table 3) even when compared to a few commercial CA, PVDF, and PES-based products (Alves et al. 2020; Yang et al. 2022), it could be assumed that the obtained membranes’ absorption capacity and subsequent remediation performance would be competitive commercially (Mohammed et al. 2018). Rejection rates against specifically Cu and/or Pb of some other commercial membranes, e.g., NF 270 (Nayak et al. 2017; Kapepula et al. 2022), polyamide-based NF (Hanif et al. 2021), NE2540-70 (Cui et al. 2014), HFN-300 AR (Kumar et al. 2021), DL, DK, NTR-7450 (Wang et al. 2007), RO X-20, NF90 (Kapepula et al. 2022), have also been reported and deemed comparable to this work’s findings. The cost-effectiveness of the synthesized CA membranes over commercial alternatives is another concern. Although cellulose isolation is time and energy-consuming, it could be considered more cost-effective than regenerating activated carbons, whose surface reactivation requires high temperature and energy (Marimuthu et al. 2023). Moreover, the offered antifouling feature in the developed MMMs could help reduce the operating cost for cleaning or replacement (El Batouti et al. 2021). Although, long-term implications (e.g., high cost, reduced flux, varying rejection rate per different filtration conditions, fouling, short service life, and weakened mechanical properties (Liu et al. 2021a, b; Li et al. 2022; Padhan et al. 2024)) might still linger, thus must be addressed. Like most similar studies, this report was based on laboratory-scale fabrication and application; thus, investigating the performance in pilot and bulk/industrial-scale is crucial in commercializing the designed membranes. The challenges include but are not limited to accessibility, renewability and recyclability, surface functionalization technological issues, biocompatibility to ensure long-term stability, standardization, and operational easiness (Vatanpour et al. 2022; Oprea and Voicu 2023; Kammakakam and Lai 2023; Padhan et al. 2024). Ultimately, aiming to comply with the textile (including ‘batik’) wastewater treatment standards, the respective national environmental regulations shall be considered. The Indonesian Ministry of Environment and Forestry Regulation No. 6 (2021) and the Malaysian-Environmental Quality Act 1974 have set maximum limits of 2.0 and 1.0 mg/L, respectively, for Cu; and 0.01 and 0.50 mg/L, respectively, for Pb (Ministry of Environment and Forestry of Republic of Indonesia 2021; Ministry of Natural Resources, Environment and Climate Change of Malaysia 2001). Therefore, it could be indicated that the resulting remediation performances were kept within the accepted values.

## Conclusion

OPEFB was chosen as raw material in synthesizing CA intended as textile dyeing wastewater treatment. ZnO and NMP in varying concentrations as filler and solvent, respectively, were explored in terms of their impact on the CA membranes’ characteristics. XRD of the prepared OPEFB-CA confirmed its high crystallinity even compared to a commercial product, as well as the fibrils web-like network by SEM–EDS. Once the OPEFB CA-NMP-ZnO membrane was prepared, its porous and smooth surface with an even dispersion of ZnO particles was also morphologically investigated, confirming the the NMP plasticizing effect. Mechanical and thermal stability improvement was also achieved. Overall, the membranes showcased a remarkable Cu and Pb remediation performance, even at high effluent concentrations. This work highlights the prospect of the use of a hefty yet notoriously underutilized biomass as a wastewater treatment alternative of the nations’ signature textile industry.

## Data Availability

Data will be provided upon request on a reasonable basis.
